# What is the clinical value of mHealth for patients?

**DOI:** 10.1038/s41746-019-0206-x

**Published:** 2020-01-13

**Authors:** Simon P. Rowland, J. Edward Fitzgerald, Thomas Holme, John Powell, Alison McGregor

**Affiliations:** 1 Department of Surgery and Cancer, Imperial College London, London, UK; 2 KPMG International, London, UK; 3 Department of trauma and orthopaedic surgery, Epsom and St Helier University Hospitals NHS, London, UK; 4 Nuffield Department of Primary Care Health Sciences, University of Oxford, Oxford, UK

**Keywords:** Public health, Therapeutics

## Abstract

Despite growing interest from both patients and healthcare providers, there is little clinical guidance on how mobile apps should be utilized to add value to patient care. We categorize apps according to their functionality (e.g. preventative behavior change, digital self-management of a specific condition, diagnostic) and discuss evidence for effectiveness from published systematic reviews and meta-analyses and the relevance to patient care. We discuss the limitations of the current literature describing clinical outcomes from mHealth apps, what FDA clearance means now (510(k)/de novo FDA clearance) and in the future. We discuss data security and privacy as a major concern for patients when using mHealth apps. Patients are often not involved in the development of mobile health guidelines, and professionals’ views regarding high-quality health apps may not reflect patients’ views. We discuss efforts to develop guidelines for the development of safe and effective mHealth apps in the US and elsewhere and the role of independent app reviews sites in identifying mHealth apps for patient care. There are only a small number of clinical scenarios where published evidence suggests that mHealth apps may improve patient outcomes.

## Introduction

The World Health Organization (WHO) Global Observatory for eHealth (GOe) defines mHealth as medical and public health practice supported by mobile devices.^1^ In total, 2.5 billion people worldwide own a mobile phone and there is huge potential for mHealth to facilitate unprecedented access to specialist clinical diagnostics and treatment advice. In the US 56% of physicians have discussed mHealth with patients and 26% have been asked about mHealth by a patient (PWC Provider Survey). Despite growing interest from both patients and healthcare providers, there is little clinical guidance on how mHealth apps should be utilized to add value to patient care, where value might include improvements in speed and accuracy of diagnosis, personalized treatment regimes, behavioral change advice, patient education or improved access to established therapies such as cognitive behavioral therapy (CBT). This article discusses the potential value of mHealth apps for patients and the challenges that clinicians face in discussing mHealth apps in clinic. Specifically, the following questions are addressed: (1) What are the different app functionalities that have clinical evidence of value added to patient care? (2) What are the challenges in taking an evidence-based approach to clinical use of mHealth apps? (3) How can medical professional societies and clinicians support patients in obtaining value from mHealth apps? (4) What value will mHealth hold for patients in the future?

## The scope of mHealth functionalities that have shown clinical evidence for patient care

One approach to categorization of mHealth apps which is used in the UK by the National Institute for Health and Care Excellence (NICE) and the National Health Service (NHS) is by their functionality.^2^ This functional classification is a basis for comparisons of levels of evidence for apps in clinical medicine. The functional categories of mHealth apps most relevant to use in clinical practice are addressed within this article and include the following:

- support clinical diagnosis and/or decision making;
- improve clinical outcomes from established treatment pathways through behavior change and enhancement of patient adherence and compliance with treatment;
- act as standalone digital therapeutics; and
- primarily to deliver disease related education.

In this section we consider the value to patients of mHealth apps in each of these categories. Other categories of mHealth apps with functionalities such as care coordination, health information portals, and those designed for rehabilitation are not included in this review.

### Diagnostics and clinical decision making through mHealth apps

It is estimated that more than 50 million people worldwide use app-based self-triage and^3^ a recent systematic review demonstrated that interactive symptom checkers are the most frequently investigated category of diagnostic app.^3^ An audit of 23 prominent symptom checker apps in a US clinic found that appropriate triage advice was given in 80% of emergency cases, which is comparable to that seen from junior doctors and senior nurses.^4^ However diagnostic accuracy was only 34% and triage advice was considered appropriate in only 55% of non-emergent cases. Diagnostic symptom checker apps targeted at specific symptoms such as hand or knee pain have been shown to frequently deliver inaccurate advice.^3^ Published evidence suggests that symptom checker apps are often risk averse and may lead to unnecessary consultations in the non-emergency setting.^3^

The other major category of diagnostic apps use algorithms to screen either photographs or data from smartphone embedded sensors. A 2017 systematic review identified 30 manuscripts investigating 35 apps most commonly intended to screen photographs for melanoma or diagnose tremor through analyses of movements.^5^ A pooled estimate of diagnostic sensitivity for such apps was reported as 82% (95% CI 0.56–0.94) and pooled specificity as 89% (95% CI 0.70–0.97).^4^ However, experts have highlighted that current evidence is not at a level to suggest that apps screening photographs could replace a clinical consultation.^6^

Despite the current limitations of diagnostic mHealth apps there is huge potential and evidence is starting to emerge to demonstrate clinically significant improvements in morbidity and mortality outcomes in specific scenarios. For example, an app designed to screen patient reported outcomes for signs of disease recurrence to allow for early re-intervention in previously treated lung cancer patients improved median survival compared to optimized standard imaging follow-up (23 months in the experimental arm vs 14.8 months in the control arm (HR 0.62, 95% CI 0.39–0.995, *P* = 0.048)).^7^ The World Health Organization already advises that digital diagnostic platforms may be valuable for patients in low- and middle-income countries where expert clinical advice is difficult to access.^8^

### Behavior change interventions through mHealth apps

Apps are potentially powerful platforms for delivery of behavior change interventions because they can improve engagement with established strategies for prevention and treatment of disease through personalized goal setting, individualized dosing reminders, and gamification.^9^ Apps that are linked to wearable devices that monitor heart rate and activity level often utilize additional behavior change techniques such as social comparison and competition, self-monitoring and review of physiological changes, and other techniques centered on social cognitive theory. A systematic review and meta-analysis of published studies investigating the effect of mobile apps to improve weight loss and physical activity outcomes demonstrated reductions in BMI of 0.43 kg/m^2^ (95% CI −0.74 to −0.13) with much greater weight loss seen when the devices were used optimally.^10^ Benefits have also been seen with the use of behavior change apps to drive improvements in glycemic control in people with diabetes through improved adherence to treatment protocols.^11^ However the challenge is to achieve sustained improvements in clinical parameters and the long-term outcomes with use of these devices is unknown.

App-based behavior change interventions are also available to address long-term non-adherence with prescribed pharmacological treatments for chronic illnesses, which is estimated to be as high as 50%.^12^ Common reasons for non-adherence that can be addressed through an app include forgetfulness, lack of understanding of side effects, and perceptions of lack of efficacy.^13^ A recent review identified 704 apps on the app store utilizing behavioral strategies to enhance medication adherence through alerts, reminders, and logs. There was positive feedback from patients on features such as customized medication regimen details and reminders; however, there were frequent technical difficulties experienced through glitches, confusing app navigation and dosage scheduling, and reminder setup inflexibility.^14^ A systematic review noted that only 4 out of 805 medication adherence apps included had associated published evidence of clinical outcomes.^15^ High-quality evidence from multicenter randomized controlled trials has recently emerged demonstrating the potential of app-based interventions to improve treatment adherence in the management of hypertension and clinical outcomes from enhanced perioperative care after elective abdominal surgery.^16,17^ While there is evidence of positive patient experiences with treatment adherence apps further research is needed to better understand how these can be utilized to add value to clinical care when prescribed alongside pharmaceuticals.

### Digital therapeutics

Digital therapeutics is a term used to describe apps that support self-management of a condition by digitalizing traditional therapies such as CBT. A systematic review of eight randomized controlled trials (*n* = 1794) investigating psychological outcomes from the use of mHealth apps to manage depression, chronic pain acceptance, insomnia severity, stress or PTSD symptoms showed variations in effect sizes (*d* = −0.13 to 1.83; 0.03–1.44) with higher engagement associated with improved outcomes.^18^ An updated meta-analysis from October 2019 identified 66 randomized controlled trials and demonstrated significantly improved clinical outcomes for management of depression, anxiety, and stress with mHealth apps vs control groups, with associated improvements in quality of life.^19^ Preliminary evidence from this analysis did not show any difference in clinical outcomes with mHealth interventions in head to head trials with face-to-face delivery of therapies. CBT-based mHealth apps have the potential to be used clinically to address a broader range of mental health conditions and are being investigated in clinical trials targeting conditions including postpartum depression, substance abuse, acrophobia, and migraine.^20–23^ Preliminary data suggest there is no significant increase in adverse events with short-term use of CBT based digital therapeutics. As with other mHealth interventions further research is required to investigate whether successful clinical outcomes are sustainable long term. Clinicians should evaluate the evidence for clinical outcomes for each digital therapeutic as popularity on the app store often does not correlate with quality of evidence.^24^

### mHealth apps that deliver disease-related education

mHealth apps can be of educational value to patients by providing structured disease and treatment-related education that is easily accessible to the user. One recent study randomized patients with knee pain to use either an educational app with structured interactive content or no intervention prior to attendance at a specialist clinic.^25^ The app improved the level of actual disease-related knowledge at clinical attendance by 52% and the perceived knowledge by 22%. There is a lack of randomized controlled trials investigating the educational value of such mHealth interventions; however, preliminary evidence does exist to support the use of specific educational mHealth interventions for patients with heart disease, inflammatory bowel disease and pediatric tonsillectomy, pediatric forearm fracture, and in patients with breast, colon, lung, prostate, and stomach cancers.^26–29^ Anecdotally such educational platforms may hold value to patients by facilitating improvements in communication with healthcare providers and enabling a level of disease understanding that supports shared decision making in clinic. Disease-related education may also hold value for patients in remote regions to support self-management. In large parts of Africa mobile phone coverage is strong and there are established programs in a number of areas. For example, a systematic review found that educational messages delivered to African women in the postnatal period may hold significant value in supporting prevention of common complications.^30^ The value of mHealth has additionally been explored as a tool to provide treatment-related education during complex treatment schedules such as those in oncology. In these situations mHealth tools have the potential to be a trusted source of relevant treatment information associated with significant improvements in patient reported quality of life.^31^

In summary there is preliminary evidence available to demonstrate the value of mHealth apps to patients in a growing number of defined clinical scenarios. Table 1 summarises different categories of mHealth apps and example clinical scenarios where evidence suggests they may add value to patient care. These conclusions are based predominantly on the findings of systematic reviews and meta-analyses of the literature which utilize the highest quality evidence available. However, variations in study populations such as language and cultural setting may impact on clinical outcomes with mHealth interventions and this should be considered when evaluating the generalizability of these findings to individual patient populations. The majority of the evidence included in this review is collected from clinical studies performed over short time periods. This is important to note particularly when considering the clinical value of mHealth interventions for behavior change apps that address chronic conditions, where the long-term value to patients remains unclear.

**Table 1:** Clinical scenarios where evidence suggests that apps may add value to patient care.

Category of mHealth App	Example clinical scenarios where mHealth apps may add value to patient care
Diagnostics and clinical decision making	Interactive symptom checkers may be used for emergency triage in areas of limited access to healthcare Screening of patient reported outcomes for recurrence of some cancers
Behavior change interventions through mHealth	Apps may support behavioral change to achieve short-term reduction of BMI for obese patients and improvement in HbA1c for mild diabetics Apps may support medication adherence in chronic disease or improve compliance and clinical outcomes from perioperative care programs
Digital therapeutics	Apps may be used to broaden access to CBT for management of insomnia and related symptoms with comparable outcomes demonstrated with traditional service provision
mHealth apps that deliver disease-related education	Apps may be used to deliver disease related education to improve communication and facilitate better patient decision making in clinic Apps may support self-management and alleviation of concerns where services are unavailable or where there are unexpected side effects from prescribed therapies

## Challenges in taking an evidence-based approach to clinical use of mHealth apps

A PubMed database search for the term “mHealth” returns more than 30,000 hits; however, our analysis identified only a handful of clinical scenarios where use of mHealth apps is supported by the highest levels of evidence. The quality of the mHealth literature is highly variable with few studies registered on clincaltrials.gov and many of the apps studied not available on the iOS or Android app store.^32^ More often than not mHealth developers simply do not have the resources available to fund large premarket prospective multicenter randomized controlled trials (equivalent to Phase III studies in the development of a pharmaceutical) and regulators currently do not insist on this. Some believe that due to lengthy time gaps from recruitment to publication, high-cost trial implementation and rigid trial protocols that a randomized controlled trial is not the optimal trial design for investigation of efficacy with mHealth apps.^33^ Furthermore, many mHealth app developers are under pressure from early stage investors to demonstrate short-term product growth. This can lead to a preference for retrospective analyses of patient-generated outcomes collected through already marketed apps.^34^ The data from such studies can be difficult for clinicians to interpret and apply in a clinical setting as users of the app are generally self-selected and their clinical outcomes may not represent the wider patient population. Another common limitation of the mHealth literature is a lack of published evidence around the usability and acceptability of individual mHealth apps for different patient populations. Such studies are particularly important for mHealth apps that are designed to stimulate behavior change because user compliance is closely linked to clinical outcomes.^35^ Retrospective analyses of patient reported outcomes may also suffer from data manipulation that may favor reporting of positive outcomes that support commercial objectives over negative findings. This can lead to significant bias.^36^ Table 2 summarizes potential limitations of retrospective analyses of patient-generated outcomes in mHealth.

**Table 2:** Limitations of retrospective analyses of patient generated outcomes in mHealth.

Few studies prospectively registered on clinicaltrials.gov
Retrospective study designs may be methodologically flawed
High risk of selection bias due to consumer self-selection of apps
Highly levels of motivation for behavior change amongst study participants leads to positive outcomes that may not be reproducible in the general population
Usability and acceptability of individual mHealth apps often not evaluated
Retrospective data analysis may favor reporting of positive outcomes that support short-term commercial objectives
Software may be frequently updated such that functionality of apps studied is not reflected in latest versions

While the methodological quality of studies across mHealth must improve, there is huge potential for unprecedented real-world data generation. Patients can be recruited for participation in clinical studies quickly and cheaply through an app, meaning the participant numbers in mHealth trials may be far superior than in traditional studies with low resource required. Once enrolled data can be collected continuously through an app on the patient’s phone, leading to large amounts of data reporting with low risk of recall bias. The involvement of commercial developers in study design can improve researchers understanding of the product, its usability and acceptability to the patient population. This is important because patient engagement may be closely related to outcomes from interventions based on behavioral modification. Greater collaboration is needed between mHealth developers and academic institutions in order to improve the mHealth literature to support evidence-based use of apps in clinic. Further research investigating the clinical benefits of mHealth apps should consider the role of the clinician in prescribing the app and how this could influence patient outcomes.

## How can clinicians support patients in obtaining value from mHealth apps?

There are thousands of health-related apps available for download from the app store but only a tiny fraction are appropriate for clinical use.^15,37^ It is challenging for clinicians and patients to identify apps that will add value to patient care partly because the standards for “approval” of apps are different across the globe. A recent systematic review identified 23 different published criteria for assessment of mHealth apps.^38^ Furthermore, many apps popular with patients are not seen as high quality by clinicians.^32,39^ It is important that medical professional societies and clinicians who recommend apps often engage patients in the decision-making process. Ideally, evaluation of an mHealth app should include assessment of the evidence for effectiveness, information governance and privacy, commissioning standards, regulatory (safety) standards, technical aspects such as the software itself, design, and interoperability standards from both patient and clinician perspectives. In the UK the Department of Health has outlined an initial “Code of Conduct” for data-driven technologies and provided NHS Digital Assessment Questions to guide commissioners.^40,41^ While these may be valuable for service commissioners, the level of technical specificity of questions mean they are not suitable for use by individual clinicians. The UK NHS Apps library approves apps based on clinical safety, data protection, security, and usability. Other online app-review sites such as iMedicalApps.com and ORCHA may also be valuable as they include clinician developed assessment criteria. In the US approval is a term often attached to apps that have gained marketing authorization through the 510k or de novo FDA clearance pathways. 510k clearance requires the manufacturer to demonstrate substantial equivalence to a previously approved medical device but the standards of evidence for clinical effectiveness are variable and different to those that apply to pharmaceuticals. In the UK and US steps have recently been taken to define minimum standards.^42^ In the US one high-profile effort to develop guidelines for the development of safe and effective mHealth apps is that of Xcertia, which is an initiative co-led by the American Medical Association, American Heart Association, and Healthcare Information and Management Systems Society (HIMSS). Xcertia aims to develop standards for the operability, privacy, security and content of mHealth apps and released draft guidance on their website in 2019. In the UK NICE, commissioned by NHS England, has used a risk and function based approach in their “evidence for effectiveness” standards, where standards of clinical evidence required for a specific product are determined by the level of risk presented by their functionality.^2^ The FDA Digital Health Software Pre-Certification program is also working to provide a standardized approach to mHealth evaluation; however, one key difference is that the Pre-Cert program focuses on approval of the software developers rather than on the software itself. The FDA argues that software products can be adapted to respond to glitches, adverse events, and other safety concerns quickly so regulation should focus on developers rather than the software itself. The 21st Century Cures act has also had a significant impact on the regulation of mHealth apps by removing the FDA's authority to request developers to withdraw apps that provide inaccurate medical information. In the US apps that make misleading claims are instead regulated by the Federal Trade Commission. Agreement is needed on a standardized approach to approval and regulation of mHealth apps in order to support clinicians and patients to identify mHealth apps that may add value to clinical care.

Counselling patients on mHealth apps is challenging and there is no evidence based standardized approach. Clinicians often lack the necessary training to counsel patients on risks that are unique to mHealth such as data privacy, which are very important to patients, and there is a lack of guidance on how to integrate digital technologies into established treatment protocols. Recently introduced data security regulations such as GDPR in Europe may further restrict the ability of clinicians to prescribe mHealth apps as many apps may not have demonstrated compliance with these standards. Current literature suggests that user factors such as a high levels of engagement with an mHealth app are frequently associated with better outcomes and clinicians may be able to support patients by setting goals that are achievable for the individual in a defined time frame with scheduled follow-up.^43^

## What value will clinical use of mHealth hold for patients in the future?

In the future it is likely that evidence-based mHealth apps will be integrated into established clinical treatment pathways, with the aim of improving outcomes from current treatments and increasing access to specialized therapies. The recent decision by the US health insurer CVS Health to fund Sleepio, a CBT based digital therapeutic, for their patients suffering from insomnia is a big step towards integration of mHealth into mainstream healthcare. There is huge potential for health data to be downloaded from mHealth apps and used to facilitate earlier detection of subclinical disease and support technology-assisted clinical decision making. We are already seeing examples of connected mhealth technologies that can accurately detect conditions such as atrial fibrillation. Ultimately mHealth technologies will have an important role to play in empowering patients to manage their own health through digitally enabled care pathways while providing additional benefits to healthcare delivery systems (Table 3). The population-level benefits of mHealth apps differ by category but include broadening of access to healthcare services, development of more cost-effective treatment pathways and improved communication with healthcare professionals. mHealth-based therapies also have significant potential to improve the sustainability of healthcare delivery in a unique way.

**Table 3:** Potential future value propositions for mHealth apps.

Population level value for each category of mHealth app	Broaden availability of services through ease of access, reduce inequalities	Cost-effective (low marginal cost, highly scalable, early detection, prevention rather than cure)	Cost-effective (reduce human resource burden on healthcare system by enabling patient-driven care)	Improve patient satisfaction through better communication with healthcare providers	Green & sustainable
Diagnostics and clinical decision making apps	✓	✓	✓	X	✓
Behavior change apps	✓	✓	✓	✓	✓
Digital therapeutic apps	✓	✓	✓	X	✓
Disease-related education apps	X	✓	✓	✓	✓

✓ yes, X no

## Conclusions

mHealth apps can be categorized into apps that support diagnostics and clinical decision making, apps that support behavior change to improve compliance with established treatment pathways, digital therapeutic apps and apps designed primarily to deliver disease-related education. Apps from each of these categories have the potential to hold value for patients when used as part of a clinical workflow; however, the level of evidence is currently only sufficient to support the use of apps in a small number of defined clinical scenarios.
